# A Genome Wide Survey of SNP Variation Reveals the Genetic Structure of Sheep Breeds

**DOI:** 10.1371/journal.pone.0004668

**Published:** 2009-03-03

**Authors:** James W. Kijas, David Townley, Brian P. Dalrymple, Michael P. Heaton, Jillian F. Maddox, Annette McGrath, Peter Wilson, Roxann G. Ingersoll, Russell McCulloch, Sean McWilliam, Dave Tang, John McEwan, Noelle Cockett, V. Hutton Oddy, Frank W. Nicholas, Herman Raadsma, for the International Sheep Genomics Consortium

**Affiliations:** 1 CSIRO Livestock Industries, St Lucia, Brisbane, Queensland, Australia; 2 United States Department of Agriculture (USDA), Agriculture Research Service (ARS), Meat Animal Research Center, Clay Center, Nebraska, United States of America; 3 Department of Veterinary Science, The University of Melbourne, Melbourne, Parkville, Victoria, Australia; 4 Australian Genome Research Centre, St Lucia, Brisbane, Queensland, Australia; 5 Johns Hopkins University, Institute of Genetic Medicine, Baltimore, Maryland, United States of America; 6 AgResearch, Invermay Agricultural Centre, Mosgiel, New Zealand; 7 ADVS Department, College of Agriculture, Utah State University, Utah, United States of America; 8 School of Meat Science, University of New England, Armidale, New South Wales, Australia; 9 Faculty of Veterinary Science, University of Sydney, Sydney, New South Wales, Australia; University of Uppsala, Sweden

## Abstract

The genetic structure of sheep reflects their domestication and subsequent formation into discrete breeds. Understanding genetic structure is essential for achieving genetic improvement through genome-wide association studies, genomic selection and the dissection of quantitative traits. After identifying the first genome-wide set of SNP for sheep, we report on levels of genetic variability both within and between a diverse sample of ovine populations. Then, using cluster analysis and the partitioning of genetic variation, we demonstrate sheep are characterised by weak phylogeographic structure, overlapping genetic similarity and generally low differentiation which is consistent with their short evolutionary history. The degree of population substructure was, however, sufficient to cluster individuals based on geographic origin and known breed history. Specifically, African and Asian populations clustered separately from breeds of European origin sampled from Australia, New Zealand, Europe and North America. Furthermore, we demonstrate the presence of stratification within some, but not all, ovine breeds. The results emphasize that careful documentation of genetic structure will be an essential prerequisite when mapping the genetic basis of complex traits. Furthermore, the identification of a subset of SNP able to assign individuals into broad groupings demonstrates even a small panel of markers may be suitable for applications such as traceability.

## Introduction

Archaeozoological evidence suggests sheep were first recruited from the wild and domesticated in the Near East approximately 8000–9000 years ago [1]. Human mediated breeding has subsequently generated specialized animals suitable for a diverse range of purposes including the production of wool, meat and milk. Since domestication, sheep have established a wide geographic range due to their adaptability to nutrient poor diets, tolerance to extreme climatic conditions and their manageable size. The result is a spectrum of phenotypically diverse populations which constitute in excess of 1400 recorded breeds [2]. To date, the genetic basis which underpins this diversity and the consequence of selection on the genetic variation present within sheep breeds has not been assayed on a genome wide basis.

The genetic history of sheep has been investigated using three major sources of genomic variation. The ovine mitochondrial genome has proven highly informative for investigations into the process of domestication, with maternal haplogroups documenting the occurrence of multiple domestication events [3]–[7]. In addition, analysis of the non-recombining region of the Y chromosome has revealed patterns of male mediated introgression during breed development [8]. Finally, autosomal microsatellites have been used extensively to estimate levels of genetic diversity [9]–[11]. Recent surveys have tested collections of animals from southern and northern Europe [12] or Europe and the Middle East [13] and facilitated analysis of genetic partitioning at a continental scale. Interestingly, southern European breeds displayed increased genetic diversity and decreased genetic differentiation compared with their northern European counterparts. This is consistent with the expectation that high genetic diversity will be maintained close to the center of domestication, and decrease with increasing geographic distance. To date, no studies have reported levels of ovine genetic diversity using autosomal variation on a global scale.

The genomic abundance and amenability to cost effective high throughput genotyping has meant that single nucleotide polymorphisms (SNP) are now the most widely used class of genetic marker in genetics. A total of 4.4 million human SNP were genotyped during phase II of the HapMap project [14] and large collections of SNP have been identified in the chicken (2.8 million [15]), dog (>2.5 million [16]), mouse (>8.2 million [17]) and cow (>60,000 [18]). In humans, genome wide association studies have utilised these markers to identify sequence variants or genomic regions associated with nearly 40 complex human diseases [19]. In domestic animals, the first genome wide association studies have demonstrated that SNP panels can be used to efficiently map Mendelian traits in dogs [20] and cattle [21]. In addition, the availability of dense SNP sets is driving investigations into the pattern of linkage disequilibrium [16], [22], [23], the dissection of QTL [24], the consequence of selection [25], [26] and genome-wide selection as a method to accelerate genetic gain in livestock [27], [28]. Knowledge concerning the extent of genetic diversity and population substructure is critical to each of these applications. For example, the contribution of hidden population stratification to the generation of false positive genome-wide association results has been demonstrated in humans [29], [30] and more recently dogs [31]. A number of studies have therefore focussed on evaluation of the genetic relatedness and substructure within human populations [30], [32] as well as breeds of dog [16] and cattle [33], [34].

The aim of this study was to develop the first set of SNP distributed across the sheep genome. This relied on re-sequencing over 2600 genomic targets which have known location within the virtual sheep genome [35]. In order to test the utility of the resulting SNP set, array based genotyping was performed to determine levels of polymorphism within 23 domestic breeds and two wild sheep species. The results indicate breeds cluster into large groups based on geographic origin, and that even a modest number of SNP can successfully identify population substructure within individual breeds.

## Results

### Re-sequencing for SNP discovery

To identify SNP, a set of 2644 genomic loci were re-sequenced using a panel of 9 individuals drawn from different breeds. A high rate of success was obtained for PCR amplification, with 2562 targets (97%) yielding fragments suitable for sequence analysis. This enabled re-sequencing of 1.226 Mb of ovine DNA in the search for polymorphisms. 6021 SNP were identified with an average density of 4.9 SNP per kb (Table 1). This is similar to the density of SNP identified between domestic lines of chickens (5.1–5.8 SNP kb ^−1^ [15]) and higher than observed between breeds of domestic dog (1.1 SNP kb ^−1^ for set 3a in [16]). SNP were identified at approximately twice the density within ovine BAC-end sequence (5.4 SNP kb ^−1^) compared with the transcribed component of the genome (ESTs; 2.5 SNP kb ^−1^, Table 1).

**Table 1:** Summary Figures for SNP Discovery

Target Type	Number a	Total kB b	SNP c	Targets with SNP	SNP kB ^−1^	Average MAF	Prop. MAF <0.1
EST	326	150	375	169	2.5	0.216	0.259
BAC end	2236	1039	5646	1637	5.4	0.235	0.169
Total	2562	1226	6021	1806	4.9	0.234	0.175

a The number of genomic targets re-sequenced

b Total nucleotides re-sequenced (kB), excluding primers

c Number of polymorphic positions identified independently using two SNP identification tools.

### SNP Type, Distribution and Minor Allele Frequency

**Figure 1:**
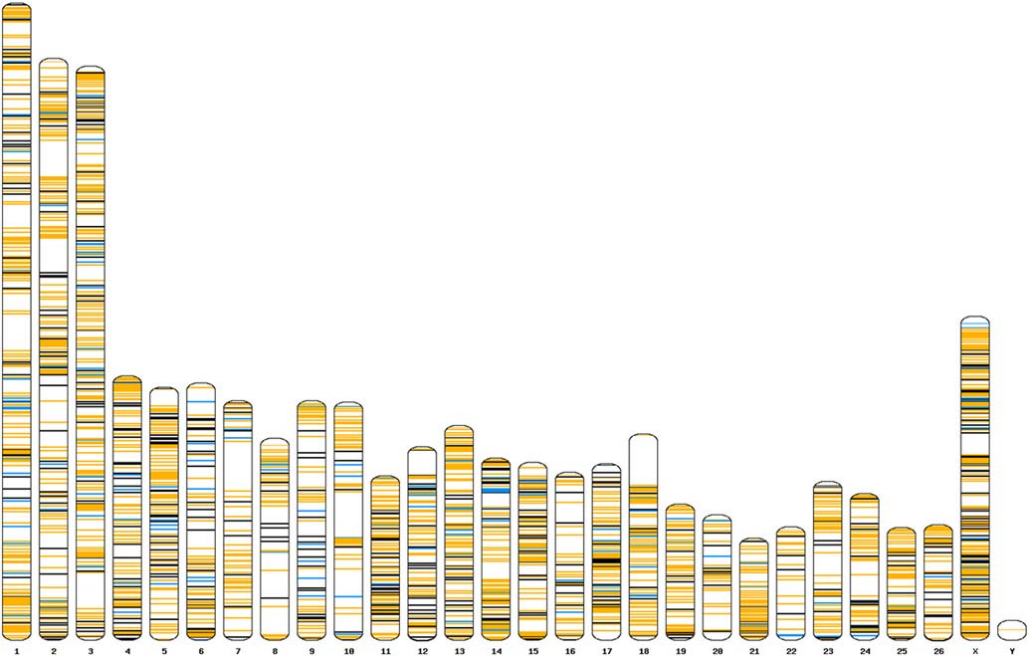
Ovine idiogram showing the distribution of targets used for re-sequencing to identify SNP. Orange bars represent genomic locations containing SNP which were formatted for array based genotyping. Blue bars represent genomic targets which yielded SNP during re-sequencing which were not included on the array. Black bars represent targets in which no SNP was identified following re-sequencing.

Analysis of the SNP set revealed 39 tri-allelic polymorphisms, 4350 transitions (α) and 1632 transversions (β), giving a mutational ratio (α/β) of 2.67. This ratio is consistent with SNP collections identified from cattle (α/β = 2.32 in 34883 SNP; Bovine HapMap consortium, personal communication William Barendse) and human (α/β = 2.36 in 10051 HSA 21 SNP [36]) but well above that observed in pig (α/β = 1.82 in 7978 cSNP [37]). The distribution of SNP in the virtual sheep genome [35] is shown in Figure 1. The average distance between re-sequencing targets containing at least one SNP was 1.03 Mb, however much larger gaps (>20 Mb) are present on chromosomes 7, 10 and 18 (gaps sizes of 22.6, 21.0 and 24.0 Mb respectively). These reflect gaps in the virtual sheep genome assembly and may represent regions of the human genome that are not represented in the sheep genome. For each SNP, minor allele frequency (MAF) was estimated from the available sequence traces and the average across all SNP was 0.234 (Table 1). The proportion of SNP with low MAF (<0.1) was noticeably higher for EST-derived SNP (0.259) compared with BAC-end derived SNP (0.169, Table 1). The distribution of MAF for both components of the genome is shown in Figure S1.

### Construction of an Ovine SNP Array and Data Filtering

To examine the utility of the SNP set for genomic research, a high throughput array was constructed and used to genotype a collection of ovine populations. The array contained 1536 SNP selected to represent the majority (952/1142) of the BAC-based comparative genomic contig (BAC CGCs) used to build the virtual sheep genome [35]. The resulting genomic distribution of SNP on the array is shown in Figure 1. A set of 413 animals were collected for genotyping which included 403 domestic animals from 23 breeds and 10 animals from two wild sheep species. The geographic origin and identity of each population used in the study is recorded in Table 2. The quality of the genotyping was high, with 95% of animals and 92% of SNP passing a set of quality control measures. These measures included removal of all data from 21 animals due to a high rate of missing genotypes and exclusion of 130 SNP due to poor assay performance. The remaining dataset contained 549,843 genotypes derived from 392 animals and 1406 SNP. Genotyping accuracy was evaluated by resequencing 8 SNPs with high MAF in 93 rams and making 739 genotype comparisons between tracefiles and chip data. Only one discordance was present and was due to allele drop-out of the PCR for sequencing. Thus, the genotyping accuracy appears to be greater than 99.8% for this experiment.

**Table 2:** Genetic Diversity within Sheep Populations

			Indices of Genetic Diversity			
Population	Code	n	Pn	Ar	pAr	He
**African Populations**						
Dorper	DOR	13	0.872	1.828	1.045	0.310
Namaqua Afrikaner	NQA	14	0.612	1.576	0.000	0.220
Red Masai	RMA	15	0.816	1.755	0.900	0.275
Ronderib Afrikaner	RDA	17	0.769	1.711	0.007	0.266
**Australian Populations**						
Merino	MER	18	0.898	1.839	1.874	0.321
Poll Dorset	DOS	20	0.855	1.792	0.010	0.300
**Asian Populations**						
Indonesian Thin Tail	ITT	8	0.730	1.730	0.046	0.273
Sumatran Thin Tail	STT	15	0.780	1.724	0.001	0.266
Tibetan	TIB	17	0.844	1.787	0.129	0.297
**European Populations**						
Charollais	CHA	14	0.836	1.797	0.362	0.308
German Mountain Brown	GMB	13	0.856	1.814	0.859	0.310
Italian Sarda	ITS	22	0.899	1.828	0.002	0.312
Scottish Blackface	SBF	15	0.857	1.814	0.011	0.315
Soay	SOA	15	0.667	1.618	0.007	0.223
Suffolk	SUF	15	0.784	1.743	0.051	0.287
**New Zealand Populations**						
Romney	ROM	18	0.864	1.814	1.507	0.314
Texel	TEX	12	0.814	1.781	0.051	0.296
**North American Populations**						
Composite	COM	16	0.870	1.825	0.084	0.319
Dorper	DOR	10	0.822	1.801	0.008	0.301
Dorset	DOS	11	0.837	1.810	0.014	0.310
Finsheep	FIN	10	0.828	1.810	0.514	0.312
Katahdin	KAT	8	0.777	1.777	0.794	0.295
Rambouillet	RAM	24	0.897	1.824	0.000	0.310
Romanov	RMV	9	0.708	1.701	0.126	0.267
Suffolk	SUF	24	0.867	1.793	0.193	0.302
Texel	TEX	10	0.803	1.781	0.017	0.294
**Wild Sheep Populations**						
Bighorn	BHS	5	0.010	1.009	0.000	0.004
Dalls Sheep	DAS	4	0.011	1.009	0.000	0.004

n number of individuals tested per population

*P*_N_ the proportion of SNP which displayed polymorphism. This is expressed as the percentage of 1406 SNP (domestic sheep) or 1397 SNP (wild sheep) which displayed multiple alleles.

*A*_R_ allelic richness

*pA*_R_ private allelic richness

*H*_E_ expected heterozygosity or gene diversity

### Minor Allele Frequency and Genetic Diversity Within Sheep Breeds

The minor allele frequency for each SNP was calculated using the genotypic data collected from the full set of domestic animals (Table 3). Examination across breeds showed 32.5% (429/1318) were polymorphic in every breed tested. In addition, the distribution of MAF revealed nearly half of the markers (45%) displayed a high degree of polymorphism (MAF ≥0.30, Table 3). This confirmed that re-sequencing a small panel of genetically diverse individuals for SNP identification resulted in a set of polymorphic markers with high utility when tested across a range of populations. Examination within breeds revealed nearly 90% of SNP displayed both alleles within breeds such as the Merino, Italian Sarda and Rambouillet (*P*_N_, Table 2). At the other extreme, only 61 and 67% of SNP were polymorphic within the Namaqua Afrikaner and Soay respectively. Averaged across breeds, 81% of SNP displayed polymorphism which indicates that the majority of identified SNP predate the radiation of the domestic breeds sampled. Examination of the variability within breeds was used to compare levels of heterogeneity between populations. This revealed that the Merino breed displayed the highest genetic diversity as measured by allelic richness (*A*_R_ = 1.839), private allelic richness (*pA*_R_ = 1.874) and gene diversity (*H*_E_ = 0.321, Table 2). Conversely the Namaqua Afrikaner was ranked lowest using each measure (*A*_R_ = 1.576; *pA*_R_ = 0; *H*_E_ = 0.220, Table 2).

**Table 3:** Minor allele frequency

Minor Allele	MAF	set1	MAF	set2	MAF	set3
Frequency	SNP	Prop.	SNP	Prop.	SNP	Prop.
0	0	0	0	0	8	0.006
>0.0 - <0.1	1051	0.175	163	0.106	189	0.143
≥0.1 - <0.2	1749	0.290	277	0.180	251	0.190
≥0.2 - <0.3	1238	0.206	373	0.243	272	0.206
≥0.3 - <0.4	1016	0.169	358	0.233	270	0.205
≥0.4 - ≤0.5	967	0.161	364	0.237	328	0.249
total	6021		1535		1318	

The number and proportion of SNP in minor allele frequency (MAF) bins is given for three sets of markers. Set 1 includes all SNP identified during the re-sequencing experiment, with MAF being calculated from sequencing data. Set 2 is the subset of markers selected for inclusion in the SNP array, for which MAF was calculated from sequencing data. Set 3 includes SNP which passed all of the quality control filters, for which MAF was calculated from genotypic data.

### SNP Genotyping Across Sheep Species

In order to assess the rate of assay conversion between species, genotyping was performed using samples from both bighorn (*Ovis canadensis,* n = 5) and thinhorn sheep (*O. dalli*, n = 4). A total of 1394 loci revealed an allele from both wild species, resulting in an assay conversion rate of 99% (1394/1406). This suggests the SNP and associated genotyping platform may be suitable for performing a phylogenetic analysis of more closely related candidate wild progenitors of domestic sheep such as the asiatic mouflon (*O. orientalis*), argali (*O. ammon*) and urial sheep (*O. vignei*). Of the SNP assays which worked across species, a total of 1355 (97%) were fixed for an allele common to both wild sheep species, while 20 markers (1.4%) displayed both alleles in at least one of the wild populations tested.

### Distance Within and Between Sheep Populations

**Figure 2:**
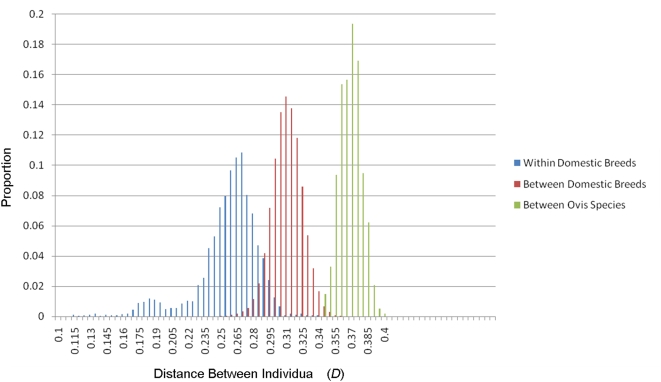
The distribution of genetic distance (*D*) between pairs of individuals. *D* was plotted separately where pairs were drawn from within the same breed (blue bars) from different breeds (red bars) from domestic sheep and one of the two species of wild sheep (*O. canadensis* or *O. dalli*, green bars).

The alleles present at each SNP were used to calculate the genetic distance (*D*) between pairs of animals. The average distance between individuals within the same breed was 0.254 (n = 3712; SD = 0.030; blue bars Figure 2). This is higher than the average obtained from analysis within 19 breeds of cattle (*D* = 0.21, SD = 0.03, Bovine HapMap consortium, personal communication, William Barendse). As expected, the average distance between individuals drawn from different breeds was higher (*D* = 0.308; n = 69441, SD = 0.014; red bars). Also as expected, the highest average distance was observed between domestic sheep and wild sheep (*D* = 0.366; n = 3447; SD = 0.010; green bars). The distribution of *D* appears normal and smooth between domestic breeds and between sheep species (Figure 2). The distribution within breed, however, has a higher standard deviation, a distinct tail toward the lower extreme and a noticeable overlap with genetic distance between sheep breeds. To investigate which pairs display the lowest distance, the *D* matrix was partitioned into breeds (Table S1). This revealed the Namaqua Afrikaner (D = 0.17±0.02) and Soay (D = 0.18±0.02) are the only two breeds which have average D<0.2, and thus make a major contribution to the tail observed in Figure 2.

### Relationship Between Breeds and the Genetic Structure of Domestic Sheep

**Figure 3:**
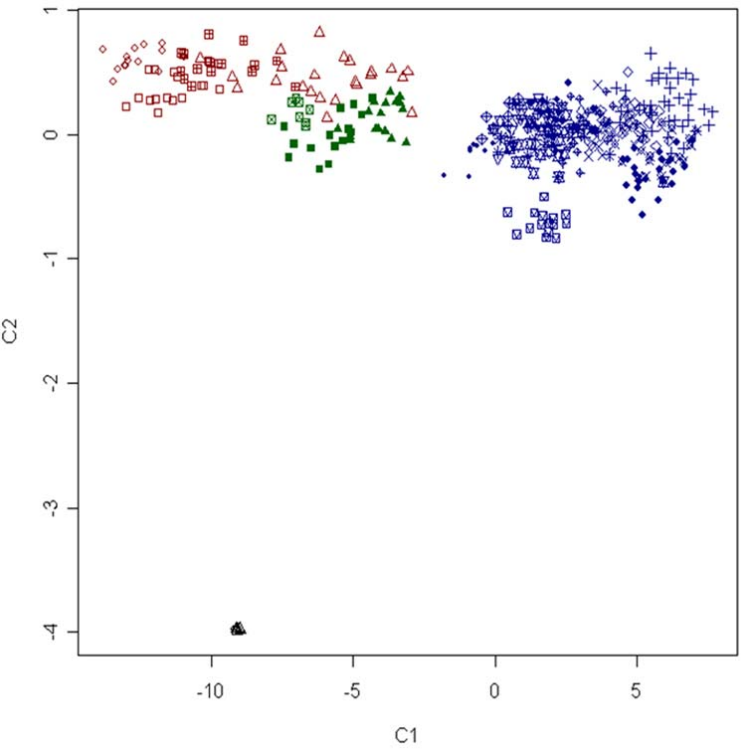
Clustering of animals from 22 breeds and two wild species of sheep, based on multidimensional scaling of genetic distance. The first (C1) and second (C2) dimensions are plotted. Animals drawn from Australia, New Zealand, Europe and North America (blue), Asia (green) and Africa (red) appear clustered according to geographic origin. Wild sheep are shown in black. Populations are represented using different characters as follows: Dorper (▵), Suffolk (+), Blackface (×), Charollais (⋄), German Mountain Brown (▿), Javanese Thin Tail (⊠), Italian Sarda (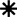), Merino (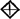), Poll_Dorset (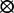), Rambouillet (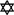), Red Masai (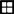), Romney (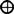), Soay (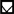), Sumatran Thin Tail (▪) , Texel (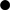) , Tibetan (▴), Finsheep (♦), Katahdin (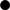), Romanov (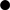), Namaqua Afrikaner (x̂), Ronderib Afrikaner (□), Composite (⋄), Bighorn (▵) and Thinhorn (x̂).

**Figure 4 pone-0004668-g004:**
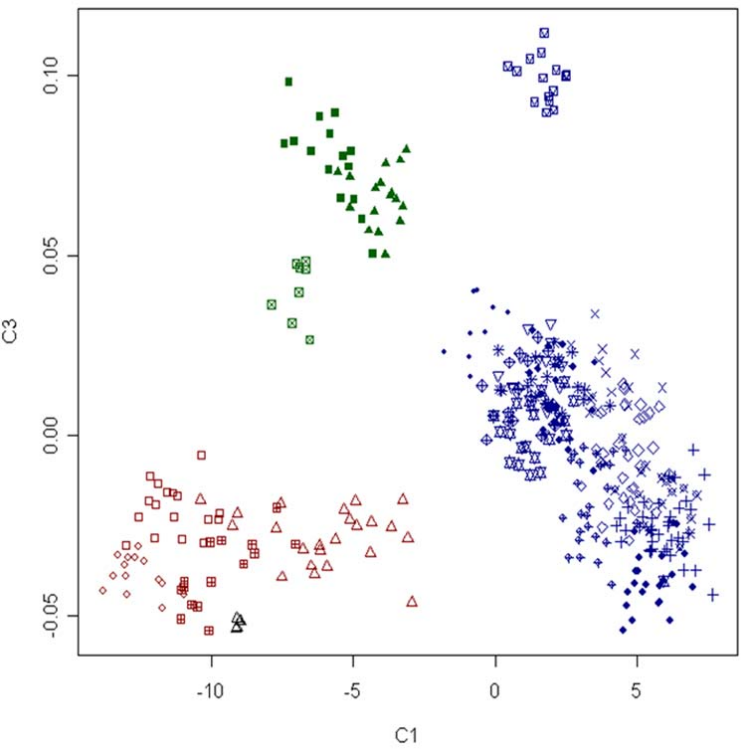
Clustering of animals from 22 breeds and two wild species of sheep, based on multidimensional scaling of genetic distance. Individuals are plotted for the first (C1) and third (C3) dimensions. Animals drawn from Asia (green) appear distinct from those drawn from Africa (red) and other countries (blue). Populations are represented using the same characters used in Figure 3 which are as follows: Dorper (▵), Suffolk (+), Blackface (×), Charollais (⋄), German Mountain Brown (▿), Javanese Thin Tail (⊠), Italian Sarda (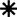), Merino (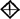), Poll_Dorset (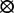), Rambouillet (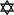), Red Masai (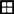), Romney (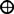), Soay (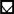), Sumatran Thin Tail (▪) , Texel (•) , Tibetan (▴), Finsheep (♦), Katahdin (•), Romanov (•), Namaqua Afrikaner (x̂), Ronderib Afrikaner (□), Composite (⋄), Bighorn (▵) and Thinhorn (x̂).

**Figure 5 pone-0004668-g005:**
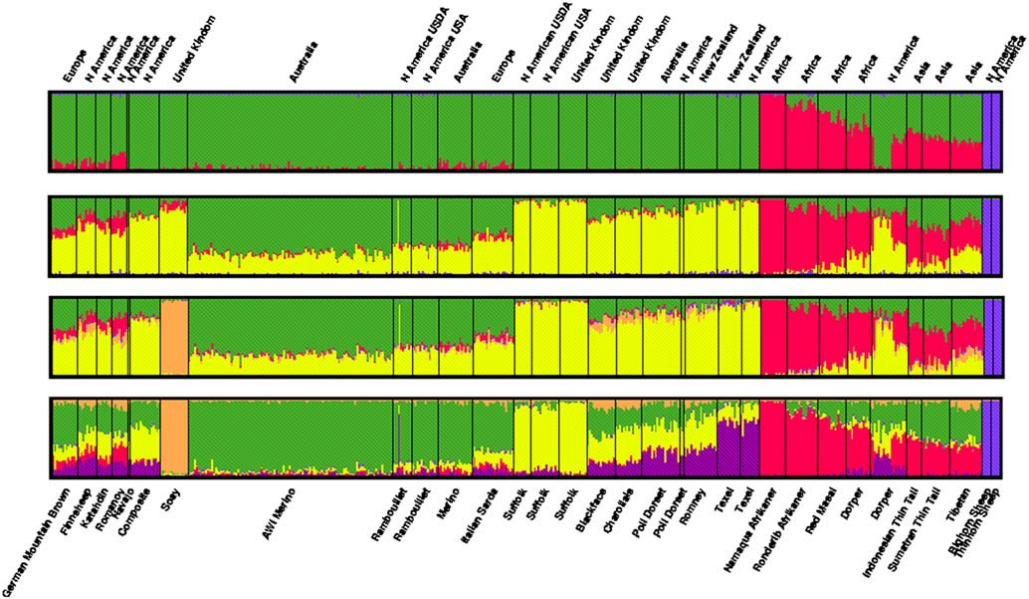
Model based clustering of 392 sheep where 3–6 sub-populations (*K*) were assumed. The geographic origin of breeds are indicated above the box plot. Individuals are represented in breed groups which are separated by vertical black lines. The breeds are given below the box plot.

Multidimensional scaling (MDS), Bayesian model-based clustering and calculation of *F*_ST_ were used to investigate the relationship between breeds and test for population substructure. The results of MDS analysis are shown in Figures 3 and 4. The first dimension (C1) separated domestic individuals into two broad non-overlapping clusters. The membership of each cluster corresponded well with the geographic origin of each breed. The first cluster contains all individuals sampled from African (NQA, RDA, RMA, DOR) and Asian breeds (STT, JTT, TIB). Individuals from the same breed occupy different areas of the cluster, indicating that substructure exists, however none of the African or Asian breeds formed a separate group. The second large cluster contained members from all breeds of European origin sampled except for the Soay which formed a separate cluster. The second dimension (C2) separated out the two species of wild sheep from domestic animals. The observation that domestic animals took a small range for C2 (−0.83–0.82) prompted plotting of C1 and C3 (Figure 4). The third dimension (C3) clustered together the four African breeds as distinct from Asian breeds but did not splinter the large cluster containing individuals from western breeds. Model based clustering was initially used to determine the minimum number of sub populations (*K*) required to explain the total sum of genetic variation observed. Figure 5 shows the distribution of individuals into clusters for *K* = 3–6. At *K* = 3, individuals from the African and Asian breeds appeared distinct from those drawn from either western breeds or wild sheep. At *K* = 4 the Soay is separated out as a distinct sub-population, at *K* = 5 the three Asian breeds (JTT, STT and TIB) can be seen as distinct from other breeds and at K = 6 a subset of African and Western breeds cluster (Figure 5). The degree of genetic differentiation between pairs of breeds measured as *F*_ST_ is presented in Table S2. The highest values (*F*_ST_>0.25) were observed between breed pairs sampled from different continents while the lowest values were observed between breeds of European origin such as the Merino and Italian Sarda (*F*_ST_ = 0.053), Suffolk and Composite (*F*_ST_ = 0.059) and the Merino and Rambouillet (*F*_ST_ = 0.060). The proportion of variation explained by geographic origin was investigated following assignment of breeds as either African (NQA, RDA, RMA, DOR), Asian (JTT, STT and TIB) or western (all others). The vast majority of SNP variation occurred within breeds (82.2%), with only 5.8% being diagnostic of differences between the three geographic groupings. The remaining component of variation was present between breeds of the same geographic region (12.0%).

### Testing for Substructure Within Sheep Breeds

**Figure 6:**
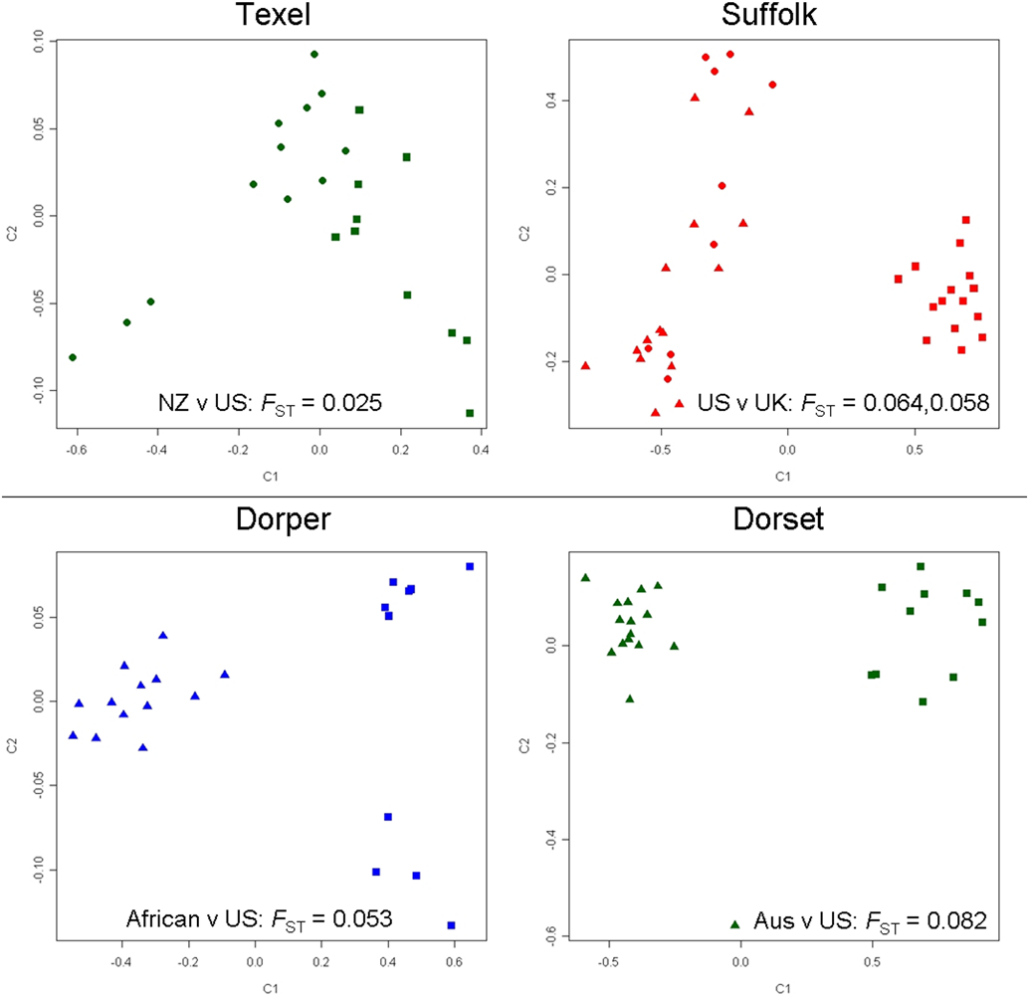
Multidimensional scaling plots for four breeds showing the genetic differentiation between geographically distinct sub-populations. The position of New Zealand (circles) and American (squares) Texels are shown in the top right panel. Two populations of American Suffolk (triangles, circles) are shown with UK Suffolks (squares) in the top left panel. African (triangles) and American (squares) Dorper are plotted in the bottom left panel while Australian Poll Dorset (triangles) and American Dorsets (squares) are shown in the bottom right panel. Note that the scale differs between panels. *F*_ST_ was calculated between each sub-population.

Separate subpopulations of the same breed were collected from different continents for the Dorper, Dorset, Suffolk and Texel (Table 2). This offered the opportunity to test if geographically distinct sub-populations could be distinguished based on genotypic data alone. MDS plots for each of the four breeds are shown in Figure 6. Australian Poll Dorset and American Dorset clustered separately and showed the highest genetic differentiation between any of the subpopulations tested (*F*_ST_ = 0.082). African and American Dorpers also clustered separately (*F*_ST_ = 0.053, Figure 6). Interestingly, the ten American Dorpers clustered into two groups which distinguished Black (n = 6) from White (n = 4) Dorper rams. Two different subpopulations of American Suffolk were each separated out from British Suffolk (*F*_ST_ = 0.064; *F*_ST_ = 0.058), although New Zealand and American Texel were indistinguishable (*F*_ST_ = 0.025).

### SNP Panels for Detecting Population Substructure

**Figure 7:**
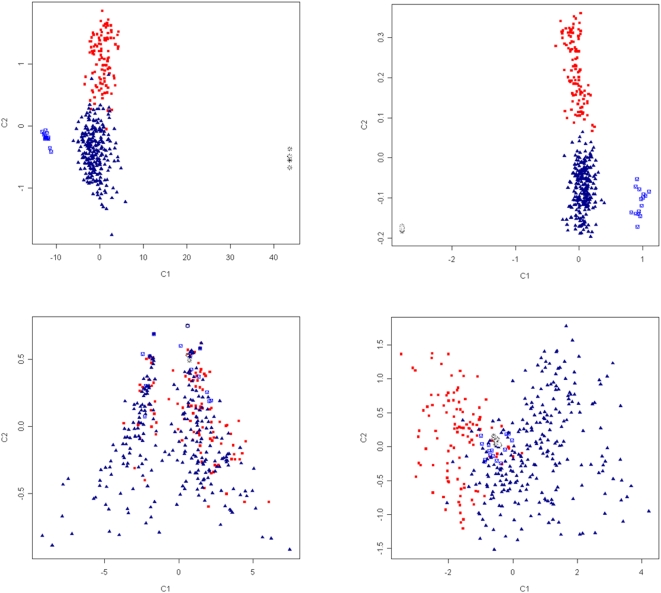
Cluster analysis of individuals from European derived breeds (blue triangles), African and Asian breeds (red squares), the Soay breed (blue patterned boxes) and wild sheep (black asterisks). Multidimensional scaling was performed using marker panels which contained either 96 (panel 1 and 3) or 384 (panel 2 and 4) SNP which were either the top (panel 1 and 2) or bottom (panel 3 and 4) ranked markers for the informativeness metric *I*_n_ [38].

The relative contribution of SNP to population assignment was estimated using the informativeness metric *I*_n_ [38]. Using *I*_n_, four marker panels were constructed which contained either the most informative or least informative SNP. Each panel was used in MDS analysis to evaluate their ability to cluster individuals into the four distinct groups observed in Figure 3. Testing revealed that while 96 of the most informative SNP were insufficient, analysis using a panel of 384 markers successfully sorted individuals into four groups (Figure 7). Conversely, analysis using the least informative SNP failed to assign individuals into discrete clusters. To identify marker attributes important for successful population assignment, the distribution of both allelic richness and private allele richness was compared between marker panels (Figure S2). Nearly half of SNP in the highly informative panel (176/384 or 45%) had near maximum allelic richness (>1.9) compared with only 7% (26/384) in the poorly informative panel. Informative SNP had higher average allelic richness (*A*_R_ = 1.82±0.22) and lower average private allelic richness (p*A*_R_ = 0.024±0.049) compared with those in the poorly informative set (*A*_R_ = 1.53±0.29; p*A*_R_ = 0.092±0.075). Together, this demonstrated a subset of markers characterised by high *A*_R_ and low p*A*_R_ can be used for population assignment.

## Discussion

We report the first genome wide set of SNP in sheep and a preliminary survey of variation across the sheep genome. The strategy for SNP identification relied on Sanger re-sequencing and a small panel of genetically diverse individuals. The resulting collection of SNP contained a mutational ratio (α/β) and genomic frequency (SNP kb^−1^) similar to datasets reported in other animal genomes. The use of a small discovery panel (n = 9) likely biased the SNP discovery process towards identification of loci with common alleles. This is supported by the finding that two thirds of SNP displayed MAF ≥0.2 when genotyped across the full set of domestic animals (Table 3). The approach also resulted in a very low false discovery rate, with less than 1% of loci displaying monomorphism across all populations (8/1318, Table 3). This likely resulted from the stringent criteria used for SNP calling which required independent identification by two analytical approaches (see Materials and Methods). Despite the high quality of the resulting data, the cost associated with Sanger re-sequencing means it is not amenable to scaling in order to generate much larger SNP sets. Fortunately it is now possible to utilise ‘next generation’ sequencing technologies which allow deep sequencing of genomic libraries to identify large numbers of SNP at comparatively low cost [18], [39].

Genome-wide association analysis offers the opportunity to identify the genomic regions and mutations which underpin disease phenotypes and production traits. To be successful, SNP are required which are both sufficiently dense as well as polymorphic within each test population. As a precursor to these activities, this study determined the proportion of markers which displayed polymorphism across a diverse range of sheep breeds. Despite using a small number of individuals for SNP discovery, over 85% of SNP with working assays were found to be polymorphic in economically important breeds such as Poll Dorset, Merino, Italian Sarda, African Dorper, Scottish Blackface, Suffolk, Romney and Rambouillet. This suggests that SNP sets identified using any of these breeds will likely have high utility for association analysis across any of the remaining breeds.

Indices of genetic diversity (*H*_E_, *A*_R_) and distance (*D*) revealed that African and Asian populations each tended to display lower variability (*H*_E_<0.3, *A*_R_<1.77) and depressed genetic distance between individuals (*D*<0.25) when compared with breeds of European origin sampled from Australia, New Zealand, Europe and North America (*H*_E_>0.3, *A*_R_>1.77, *D*>0.25; Tables 2 and S1). It is important to note this trend was accompanied by a generally lower proportion of markers displaying polymorphism in African and Asian breeds (Table 2). Fewer variable markers will serve to reduce population measures such as *H*_E_, *A*_R_ and *D*, meaning African and Asian breeds may not necessarily contain less genetic variability. A paucity of existing data from African and Asian animals makes calibration of this finding difficult, however a recent and comprehensive survey of Ethiopian sheep revealed them to carry higher levels of diversity (*H*_E_ = 0.71, *A*_R_ = 6.79 [40]) than found in northern European breeds (*H*_E_ = 0.67, *A*_R_ = 5.09 [10]). This suggests that other factors such as non-representative population sampling, differences in effective population size or an ascertainment bias in SNP discovery may have contributed to the findings in the current study. One clear example is the low genetic diversity observed in the Soay (*H*_E_ = 0.223, *A*_R_ = 1.618, *D* = 0.184, Tables 2 and S1). These animals were sampled from a group of isolated Scottish islands and have a small effective population size [41] and low levels of diversity when assayed using microsatellite markers [12]. The factors resulting in similarly low diversity in some African (NQA, RDA) and Asian breeds (JTT, STT) is less clear and leaves open the possibility that ascertainment bias in the SNP discovery process may be responsible. A very strong bias would be expected to generate an excess of low MAF SNP in breeds not represented during the SNP discovery process. This was examined by excluding monomorphic SNP within each breed and comparing the MAF profile of the remaining loci. Figure S3 shows no significant differences were observed, however some degree of ascertainment bias may still be in operation. In an effort to avoid such bias, four of the nine animals used in the re-sequencing panel were drawn from non European derived breeds. Despite this, a dedicated SNP discovery effort may be required using exclusively African and Asian breeds.

In order to examine the degree of phylogeographic structure in domestic sheep, the distribution of SNP variation was examined as a function of both breed membership and geographic origin. The finding that only 5.8% of variation was partitioned between geographic groupings and 82.2% was resident within breeds indicates sheep have the weakest phylogeographic structure of any domestic species examined to date. This is consistent with a microsatellite based study which found less than one percent of variation was explained by grouping 29 breeds into seven geographic regions across the Near East and Europe [12]. Analysis of mtDNA haplotypes in sheep [42] and goat [43] have found similar results, prompting speculation that the small size and versatility of sheep and goats have enabled their transportation and subsequent introgression in concert with human migration [43], [44]. The findings presented in the current study clearly support the conclusion that high levels of introgression have occurred, especially among western breeds. For example, cluster based analysis revealed the majority of western breeds form a single cluster (Figures 3 and 4) and estimation of the genetic distance revealed that some sheep are more closely related to individuals from a different breed than to other members of their own breed (overlapping distribution of D in Figure 2). Taken together, the results reveal sheep breeds share high levels of genetic similarity which is consistent with their short history. Most western breeds were formed within the last 200 years and while most have undergone selection, few appear to have been maintained as truly isolated populations.

While low, the degree of population structure was still sufficient to sort individuals into groups which displayed concordance with known breed history and broad geographic classification (Figure 3 and 4). The largest cluster contained all of the European breeds tested along with populations from Australia, New Zealand and North America. The finding that these geographically separate populations are genetically similar is entirely consistent with the recorded history of the breeds tested. The Merino, Poll Dorset, Romney and Texel were all originally developed in either England or continental Europe prior to importation into Australia and New Zealand during the 18^th^ and 19^th^ century [45], [46]. Similarly the Dorset, Finnsheep, Rambouillet, Romanov and Suffolk populations from North America each have an established European origin [47]. Conversely, indigenous breeds of African (Red Masai, Namaqua Afrikaner and Dorper) and Asian sheep (Javanese Thin Tail and Tibetan) were genetically distinct from those of European origin and formed separate clusters (Figure 3–6). In addition to this broad level classification, the amount of genetic substructure was also sufficient to detect stratification beneath the level of breed in some, but not all, of the populations tested. Specifically, geographically distinct subpopulations within each of three breeds were clearly distinguished using genotypic data alone (Figure 6). This opens the possibility that an informative SNP panel can be used within an industrial setting for tracing the geographic origin of animal products such as meat. This is likely to be important given the non-uniform prevalence of diseases such as scrapie, blue tongue and foot and mouth disease. To create a tool for industrial application, a SNP panel was identified which successfully reconstituted the clustering of individuals achieved using the full set of markers (Figure 7). The size of the panel (384 SNP) ensures it is configured for a commercially available genotyping platform. In addition, it is composed of SNP which have high allelic richness and low private allelic richness (Figure S2) which indicates that differences in allele frequency provide the basis for assignment of individuals into discrete populations. It is therefore likely that the panel may be used for assignment of parentage. This has proven successful in cattle [48] and the observation that approximately one third of markers were polymorphic in all of the breeds tested indicates that a carefully selected subset of SNP should have utility in almost any sheep breed.

## Materials and Methods

### SNP Discovery

A set of 2644 genomic loci were selected for amplification and re-sequencing in an attempt to identify SNP. This included 350 targets associated with the exons of genes (ESTs) and 2294 targets drawn from a library of BAC end sequences (BES) after screening for repeat sequences and location in the genome. BES targets were selected to be approximately one third and two thirds of the way along each BAC comparative genomic contig in the virtual sheep genome [35]. A small number of additional BAC end sequences were included to resolve ordering in a small number regions with high uncertainty. Primers were designed to amplify fragments with an average length of 508 bp. EST based primers were positioned within exons longer than 650 bases. Genomic DNA from a diversity panel was used for re-sequencing which consisted of one individual drawn from each of the following nine divergent breeds: Awassi, Gulf Coast Native, Katahdin, Lacaune, Merino, Poll Dorset, Red Masai, Romney and Texel. PCR amplification was performed in 10 µl before 50–100 ng was sequenced using BDTv3.1 chemistry and an ABI Prism 3730 (Applied Biosystems) DNA sequencer at the Australian Genome Research Facility. A Beckman Coulter Biomek NX 384 liquid handler was used for large volume manipulations (4–20ul) and Deerac Equator GX-8 liquid handler was used for small volume manipulations (0.5–2ul). Polymorphic bases were identified using SNPdetector [49] and polyphred v 5.01 [50]. The 6021 SNP reported represent all of the nucleotide positions independently identified as polymorphic using both prediction programs.

### Data Access

Information for all SNP is accessible through the virtual sheep genome browser at http://www.livestockgenomics.csiro.au/perl/gbrowse.cgi/vsheep1.2/. Figure S4 illustrates the data available using a 5 Mb region of chromosome 13. Clicking on individual SNP provides access to the sequence trace files, sequence alignments, the primers used for analysis, the genomic position and minor allele frequency data for each SNP. SNP have been deposited into dbSNP with accession numbers ss73688717 - ss76881533.

### Design of the ovine 1536 SNP array and genotyping

For each of the 6021 SNP identified, the variant position and repeat masked flanking sequence (≥70 bp on each side) was used to calculate design scores for the golden gate assay (performed by Illumina). A total of 1535 SNP were selected for inclusion on the array to satisfy both assay design score (≥0.6) and genomic location by including markers on each contig of the virtual genome. A single SNP located within the male specific region of the ovine Y chromosome was also included (*oY1* [51]). Genotyping was performed on genomic DNA (75–150 ng/µl) using the highly multiplexed bead array assay [52] at the Johns Hopkins SNP Center (http://snpcenter.grcf.jhmi.edu/). Population samples (Table 2) were collected to ensure individuals were as unrelated as possible. A single downloadable file containing the genotypic data derived from each animal is available at http://www.sheephapmap.org/28pops_1406loci.arp.zip.

### Estimates of the Genetic Diversity

Estimates within each population of the proportion of polymorphic markers (*P*_N_), allelic richness (*A*_R_) and private allelic richness (p*A*_R_) were determined using HP-RARE v1.0 [53], while estimates of gene diversity (*H*_E_) were obtained using Genetic Data Analysis v1.0 [54]. FSTAT 2.9.3.2 (http://www2.unil.ch/popgen/softwares/fstat.htm) was used to evaluate population relatedness using pair-wise estimates of *F*_ST_. The partitioning of SNP variation was conducted using an analysis of molecular variance (AMOVA) as implemented in Arlequin v3.01 [55]. A hierarchical grouping was imposed on the data to examine the proportion of variance residing at three levels: 1) within breeds 2) between breeds within the same geographic region and 3) between geographic regions. Regions were defined as African, Asian or western as described in the text.

### Allele Sharing and Distance

Genetic distance between all pair-wise combinations of individuals (*D*) was calculated as one minus the average proportion of alleles shared, as described by [56]. The average proportion of alleles shared was calculated as (IBS2+0.5*IBS1)/N, where IBS1 and IBS2 are the number of loci which share either 1 or 2 alleles identical by state (IBS), respectively, and N is the number of loci tested. This was performed using PLINK v 1.01 (http://pngu.mgh.harvard.edu/purcell/plink/), where the average proportion is reported as Dst. A total of 1315 SNP were used, following pruning of SNP which had MAF <0.01 and/or greater than 10% of missing genotypes. The distribution of D was plotted separately where the pairs of individuals were drawn a) from within the same breed b) from different breeds or c) from *O. aries* and one of the two species of wild sheep (*O. canadensis* or *O. dalli*).

### Analysis of Genetic Structure

Multidimensional scaling (MDS) analysis used a total of 1317 SNP following removal of loci with missing genotype rate of >0.1 or MAF <0.01. An IBS matrix of distance (*D*) was constructed containing each pair-wise combination of all 392 individuals. Both SNP pruning and calculation of *D* was performed using PLINK (http://pngu.mgh.harvard.edu/purcell/plink/). Classical (metric) MDS analysis was then applied to explore the similarities in the matrix. The – cluster and – mds-plot functionality implemented in PLINK was used without the addition of any constraint. It should be noted that when MDS is based on *D* it is numerically identical to principal components analysis [57]. The extent of population substructure was explored using structure v 2.2 [58]. All 392 animals were used and three replicate runs were performed for K = 2–10, 15, 20, 25 and 30 where K is the number of subpopulations. In each case, the admixture model was chosen and the runs were carried out using 20000 MCMC burnin replications followed by a 30000 run length. The averaged likelihood at each *K* [ln Pr(X | *K*) or Ln(K_n_)] and its variance between replicates was used to search for the most likely number of subpopulations. The likelihood approached an asymptote and the variance between runs increased approaching *K* = 10 (Ln(K_2_) = −6.847×10^5^±24.2; Ln(K_3_) = −6.74×10^5^±317.3; Ln(K_4_) = −6.674×10^5^±285; Ln(K_5_) = −6.602×10^5^±35.4; Ln(K_6_) = −6.558×10^5^±161.2; Ln(K_7_) = −6.519×10^5^±659.8; Ln(K_8_) = −6.478×10^5^±136.5; Ln(K_9_) = −6.464×10^5^±440.2; Ln(K_10_) = −6.587×10^5^±1254.7). At values of K>15, the likelihood dropped dramatically (Ln(K_20_) = −6.940×10^5^; Ln(K_25_) = −6.934×10^5^ Ln(K_30_) = −7.140×10^5^) suggesting an optimal value of *K*<10. The solutions for K = 3–6 were visualised using distruct ver 1.1 [38]. The informativeness for assignment (*I*_n_) was estimated using Infocalc ver1.1 [59]. Individuals were first classified as either 1) African and Asian; 2) western excluding the Soay; 3) Soay or 4) wild sheep to represent the four major clusters observed in Figure 3 before genotypic data was used to estimate *I*_n_ for all markers (range 0–1). The highest ranking SNP defined panels 1 (96 SNP) and 2 (384 SNP) while the lowest ranked SNP with non-zero *I*_n_ defined panels 3 (96 SNP) and 4 (384 SNP). MDS using each marker panel was as described previously.

### International Sheep Genomics Consortium

**Wes Barris**, CSIRO Livestock Industries, St Lucia, Brisbane, QLD 4067, Australia; **Steve C Bishop**, The Roslin Institute and Royal (Dick) School of Veterinary Studies, University of Edinburgh, Roslin, Midlothian, EH25 9PS, UK; **David Coltman**, Department of Biological Sciences, University of Alberta, Edmonton AB T6G 2E9, Canada; **Allan Crawford**, AgResearch, Invermay Agricultural Centre, Mosgiel, Private Bag 50034, New Zealand; **André Eggen**, INRA, UR339 Laboratoire de Génétique Biochimique et Cytogénétique, F-78350 Jouy-en-Josas, France; **Georg Erhardt**, Institut für Tierzucht und Haustiergenetik Justus-Liebig-Universität Gießen, Ludwigstraße 21 B, 35390 Gießen, Germany; **Robert Forage**, SheepGenomics 165 Walker St North Sydney NSW 2060 Australia; **Olivier Hanotte**, International Livestock Research Institute (ILRI) PO Box 30709 Nairobi, Kenya; **Peter Hunt**, CSIRO Livestock Industries, Armidale, NSW 2351, Australia, **Han Jianlin**, CAAS-ILRI Joint Laboratory on Livestock and Forage Genetic Resources, Institute of Animal Science, Chinese Academy of Agricultural Sciences (CAAS), Beijing 100193, China; **Kui Li**, Institute of Animal Science, Chinese Academy of Agricultural Sciences, Beijing 10094 PR China; **Paolo Ajmone Marsan**, Via Emila Parmense, 84 Universita Cattolica del Sacro Cuore, 29100 Piacenza Italy; **James E. Miller**, Department of Pathobiological Sciences, School of Veterinary Medicine, Louisiana State University, Baton Rouge, LA 70803, USA; **Josephine Pemberton**, Institute for Evolutionary Biology, University of Edinburgh, Edinburgh EH9 3JT United Kingdom and **Laurent Schibler**, Laboratoire de Génétique biochimique et de Cytogénétique, INRA-CRJ, Jouy-en-Josas, France as part of the International Sheep Genomics Consortium.

## Supporting Information

Table S1 Genetic distance within each sheep population.
(0.02 MB DOC)

Table S2 Genetic differentiation between population pairs measured using FST.
(0.07 MB DOC)

Figure S1 Minor allele frequency (MAF) distribution for SNP identified from either expressed sequence tags (n = 375) or BAC end sequence (n = 5646). The proportion of total SNP in each MAF category is shown.
(0.12 MB TIF)

Figure S2 Distribution of allelic richness (top) and private allelic richness (bottom) for SNP panels 2 and 4 (refer to Figure 7) which have either high informativeness (red) or low informativeness (green) for population assignment. Allelic richness ranges between 1 and 2 for biallelic SNP while private allelic richness ranges from zero to 1. Informative SNP tend to have high allelic richness and low private allelic richness.
(0.21 MB TIF)

Figure S3 Minor allele frequency (MAF) distribution between breeds was used to test for the presence of strong ascertainment bias. The breeds shown were either present (MER, RMA) or absent (NQA, SOA and STT) from the SNP discovery panel and displayed either a low (NQA, SOA) medium (STT) or high (MER, RMA) proportion of polymorphic loci (Pn, Table 2). Breed abbreviations are given in Table 2. For each population, monomorphic loci were excluded before MAF was calculated using the remaining SNP. Severe ascertainment bias should result in an excess of low MAF SNP in breeds not represented in the discovery process, however no significant differences (p>0.05) were observed between any pairwise combination of breed specific MAF profile.
(0.17 MB TIF)

Figure S4 SNP data available at the virtual sheep genome browser. The top half of the figure illustrates the genomic location of targets used for re-sequencing to identify SNP. The bottom half of the figure illustrates the information available for one target (DU324092). This includes SNP location, flanking sequencing and type of SNP. The virtual sheep genome browser is available at http://www.livestockgenomics.csiro.au/perl/gbrowse.cgi/vsheep1.2/.
(0.31 MB TIF)
