# Supplementary material for: A Genome Wide Survey of SNP Variation Reveals the Genetic Structure of Sheep Breeds
Source: PLoS One. 2009 Mar 3;4(3):e4668. doi: 10.1371/journal.pone.0004668 (PMC2652362; doi:10.1371/journal.pone.0004668)
Supplement: Table S1 — Genetic distance within each sheep population. (0.02 MB DOC) [file pone.0004668.s001.doc]

**Table S1**

Average (*D*) and standard deviation (*D* sd) distance between pairs of animals within each sheep population, based on n pairs.
