# Supplementary material for: A Genome Wide Survey of SNP Variation Reveals the Genetic Structure of Sheep Breeds
Source: PLoS One. 2009 Mar 3;4(3):e4668. doi: 10.1371/journal.pone.0004668 (PMC2652362; doi:10.1371/journal.pone.0004668)
Supplement: Table S2 — Genetic differentiation between population pairs measured using FST. (0.07 MB DOC) [file pone.0004668.s002.doc]

**Table S2**

Genetic differentiation between population pairs measured using *F*_ST_. Three letter population codes are defined in Table 2. The highest and lowest ten *F*_ST_ values are highlighted in red and green respectively.
