# Supplementary figures and images for: A Genome Wide Survey of SNP Variation Reveals the Genetic Structure of Sheep Breeds
Source: PLoS One. 2009 Mar 3;4(3):e4668. doi: 10.1371/journal.pone.0004668 (PMC2652362; doi:10.1371/journal.pone.0004668)

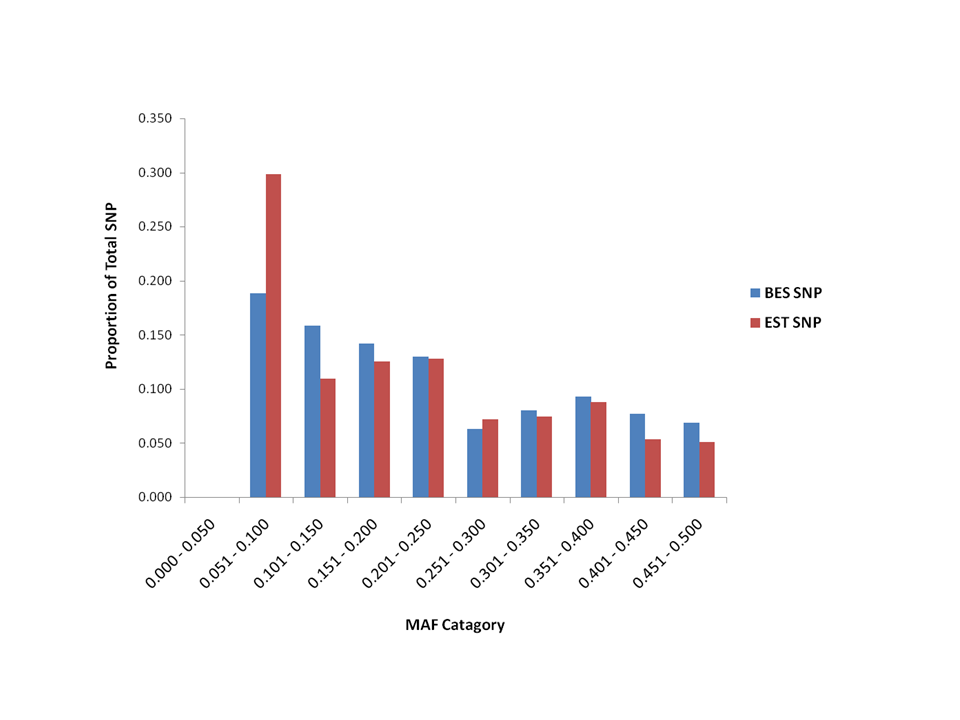

Supplement: Figure S1 — Minor allele frequency (MAF) distribution for SNP identified from either expressed sequence tags (n = 375) or BAC end sequence (n = 5646). The proportion of total SNP in each MAF category is shown. (0.12 MB TIF) [file pone.0004668.s003.tif]

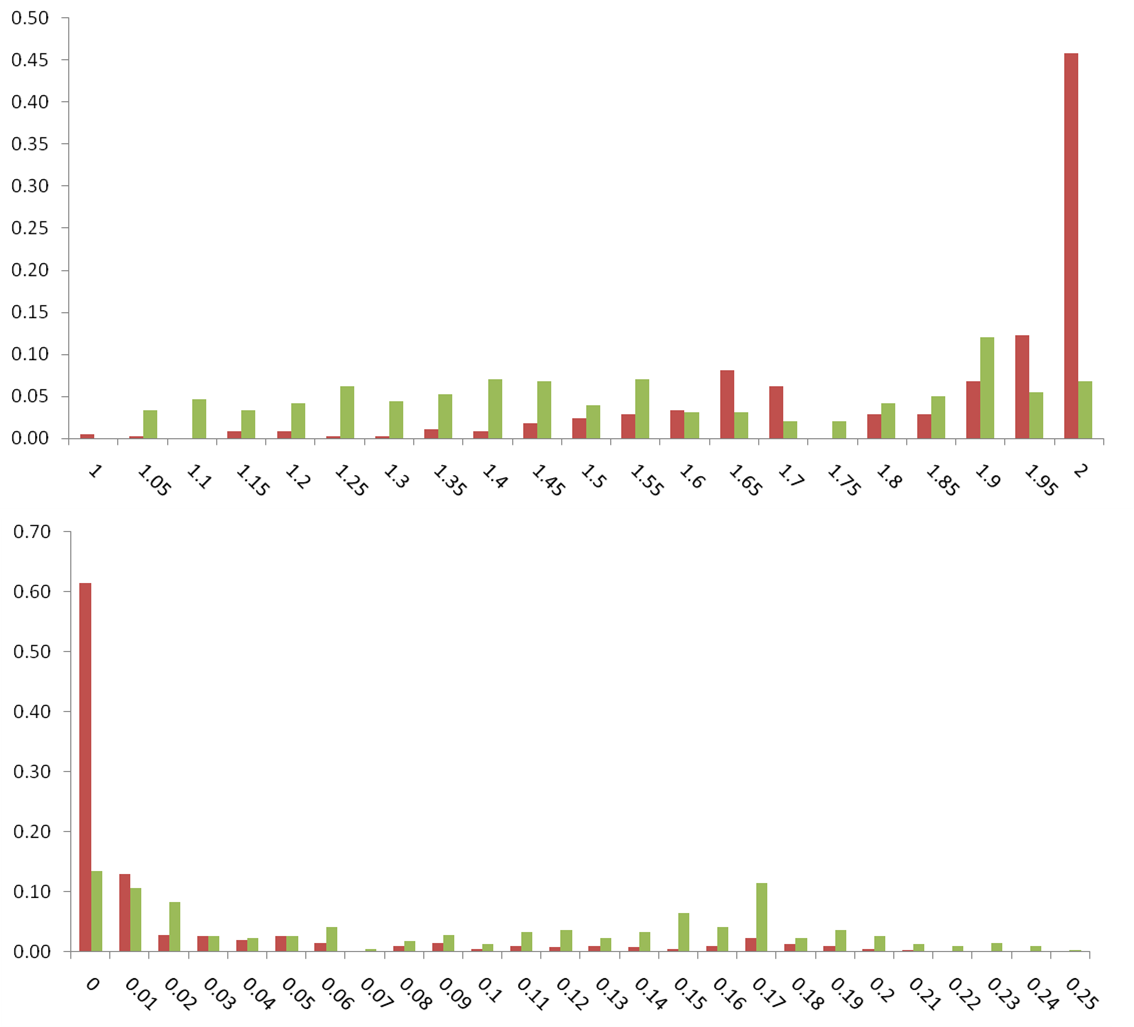

Supplement: Figure S2 — Distribution of allelic richness (top) and private allelic richness (bottom) for SNP panels 2 and 4 (refer to Figure 7) which have either high informativeness (red) or low informativeness (green) for population assignment. Allelic richness ranges between 1 and 2 for biallelic SNP while private allelic richness ranges from zero to 1. Informative SNP tend to have high allelic richness and low private allelic richness. (0.21 MB TIF) [file pone.0004668.s004.tif]

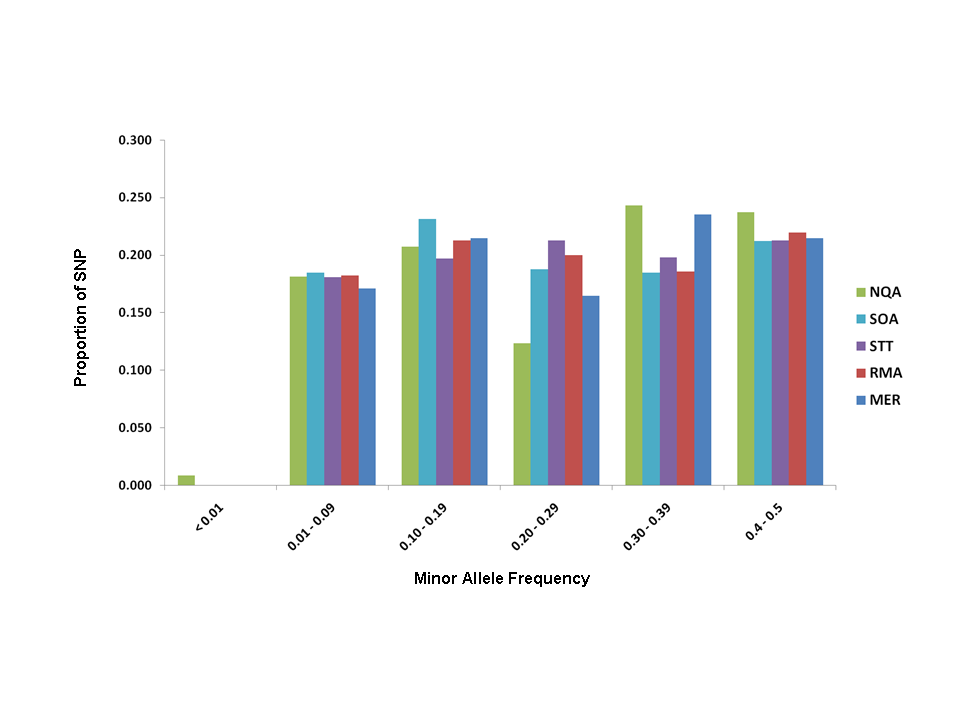

Supplement: Figure S3 — Minor allele frequency (MAF) distribution between breeds was used to test for the presence of strong ascertainment bias. The breeds shown were either present (MER, RMA) or absent (NQA, SOA and STT) from the SNP discovery panel and displayed either a low (NQA, SOA) medium (STT) or high (MER, RMA) proportion of polymorphic loci (Pn, Table 2). Breed abbreviations are given in Table 2. For each population, monomorphic loci were excluded before MAF was calculated using the remaining SNP. Severe ascertainment bias should result in an excess of low MAF SNP in breeds not represented in the discovery process, however no significant differences (p>0.05) were observed between any pairwise combination of breed specific MAF profile. (0.17 MB TIF) [file pone.0004668.s005.tif]

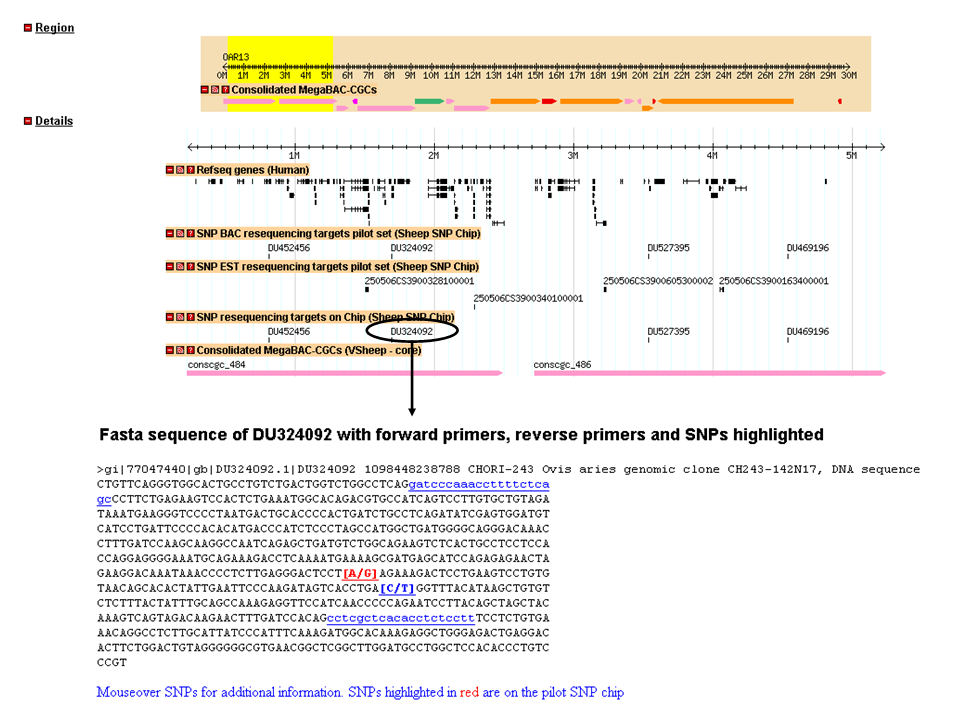

Supplement: Figure S4 — SNP data available at the virtual sheep genome browser. The top half of the figure illustrates the genomic location of targets used for re-sequencing to identify SNP. The bottom half of the figure illustrates the information available for one target (DU324092). This includes SNP location, flanking sequencing and type of SNP. The virtual sheep genome browser is available at http://www.livestockgenomics.csiro.au/perl/gbrowse.cgi/vsheep1.2/. (0.31 MB TIF) [file pone.0004668.s006.tif]
